# A New Therapeutic Approach for Dystussia and Atussia in Neurogenic Dysphagia: Effect of Aerosolized Capsaicin on Peak Cough Flow

**DOI:** 10.1007/s00455-022-10439-z

**Published:** 2022-04-16

**Authors:** Eliane Lüthi-Müller, Jan Kool, Veit Mylius, Paul Diesener

**Affiliations:** 1 Department of Neurology, Kliniken Valens, Taminaplatz 1, 7317 Valens, Switzerland; 2 Clinic for Rehabilitation, Center for Parkinson’s Disease, 8588 Zihlschlacht, Switzerland; 3 Department of Neurology, Philipps University of Marburg, 35037 Marburg, Deutschland

**Keywords:** Aspiration pneumonia, Neurogenic dysphagia, Dystussia, Tracheobronchial clearance, Aerosolized capsaicin, Peak cough flow

## Abstract

Swallowing and cough are crucial components of airway protection. In patients with neurogenic dysphagia (ND), there is a high prevalence of dystussia (impaired cough) and atussia (absence of cough). As a result, the ability to detect and remove aspirated material from the airway decreases, exacerbating the sequelae associated with ND, including aspiration pneumonia, a leading cause of mortality in ND. This controlled intervention study aimed to quantify the cough response to aerosolized capsaicin (AC) in patients with ND and assess the potential of AC as a therapeutic tool in treating ND-related dystussia and atussia. Furthermore, we propose a novel application method that enables AC treatment to be performed at home. Spirometry was used to measure peak cough flow (PCF) of voluntary cough (cough on command) and reflexive cough (cough secondary to pharyngeal exposure to AC) in 30 subjects with and 30 without ND. The capsaicin aerosol was generated by adding 1–10 drops of liquid cayenne extract (1.5–2% capsaicin) to 100 mL carbonated water (0.00075–0.001% to 0.0075–0.01% capsaicin). Voluntary PCF in the ND group was significantly lower than in the control group (*p* < 0.001), while there was no significant difference in reflexive PCF (*p* = 0.225). Within the ND group, reflexive PCF was significantly higher than voluntary PCF (*p* = 0.001), while in healthy controls, reflexive PCF was significantly lower (*p* < 0.001). The data show that AC increased the tracheobronchial clearance efficacy in ND patients with dystussia and atussia, as it enabled subjects to access their individual cough potential, which is present, but inaccessible, due to neurological disorder.

## Background

Airway protection is the ability to prevent aspiration of foreign or endogenous material into the airway (aspiration prevention) and, if aspiration occurs, detect and remove the material (aspiration correction). It includes a continuum of highly coordinated behaviors, ranging from swallowing (primary preventative behavior) to cough (primary corrective behavior) [1, 2]. Deficits in airway protection are associated with decreased quality of life and increased risk of immediate or delayed sequelae, such as choking, asphyxiation, malnutrition, dehydration, and aspiration pneumonia (AP). The anatomic focal point of aspiration prevention is the pharynx, which bifurcates in the caudal part into the esophagus posteriorly and larynx anteriorly, and serves as a conduit for both air and various bolus materials [3]. Essential in mitigating the aspiration risk inherent in this cross-system use is adequate pharyngeal swallowing function, i.e., the ability to move the bolus through the pharynx around the larynx and into the esophagus without material entering the trachea and bronchi [4]. Pharyngeal swallowing is a sensorimotor behavior that includes a precisely patterned sequence of motor events, e.g., glottal closure, arytenoid adduction, and epiglottal folding. It requires coordination with breathing (e.g., apnea during swallowing) and other processes, such as vocalization [3]. It is physiologically complex and prone to malfunction. Therefore, aspiration is not uncommon, even in healthy populations. However, severe complications or death are relatively rare due to the aspiration correction mechanism, whose primary behavior is cough. Cough removes aspirated material by generating high-velocity shearing forces in larger airways and squeezing actions in smaller airways [1]. Induction occurs by cortically mediated voluntary activation, on command, or by conscious effort (voluntary cough), or secondary to stimulation of multiple types of sensory nerves innervating the airways in different densities (reflex cough) [5].

The debate over the exact subtypes of primary sensory neurons participating in cough generation and modulation is ongoing. In their dual-sensory neuron model, Canning et al. identify chemo-sensitive, unmyelinated C-fiber nociceptors and myelinated Aδ fibers, which are insensitive to most chemical mediators (except rapid changes in pH), but highly sensitive to mechanical stimuli [6–8]. Cough-inducing, or tussigenic, stimuli include aspirated foreign or intrinsically produced materials (e.g., food, liquids, sputum, gastric content), inhaled particulate matter, inflammatory mediators (e.g., bradykinin), and irritants (e.g., citric acid, aerosolized capsaicin) [5]. In the past two decades, our understanding of airway protection neurophysiology has evolved from swallowing and cough as separate reflexive behaviors expressed by separate central pattern generators (CPGs) in the brainstem to a continuum of highly coordinated behaviors intimately connected via a sensorimotor control network with shared neural substrates in the brainstem as well as in cortical and subcortical regions [2, 9–11]. Given the potential of shared neural circuitry, it is reasonable to hypothesize that disease and damage have distributed effects [12]. A consequence of this may be the high prevalence of dystussia (impaired cough) and atussia (absence of cough) in neurogenic dysphagia (ND).

The airway protection deficits associated with the concurrence of swallowing and cough impairments can have deleterious effects on patients’ health and quality of life [13–18]. Of particular concern is AP, a leading cause of mortality in ND [19, 20]. Historically, AP has been attributed almost solely to dysphagia-related aspiration [7]. Accordingly, AP prevention strategies have focused mainly on preventing aspiration by improving swallowing safety. While aspiration is undoubtedly a prerequisite for AP pathogenesis, a growing body of literature identifies impaired cough function as a critical factor and, consequently, a clinically relevant therapeutic target.

The absence of reflex cough in response to aspiration is termed silent aspiration [21]. Dysphagic patients with silent aspiration are at a significantly increased risk of developing pneumonia [22, 23]. In comparison, dysphagic patients with adequate cough function rarely develop AP [24]. Nevertheless, protussive therapies for this patient population are minimal [25]. Thus, we investigated methods to treat dystussia and atussia in ND.

Earlier studies have attributed cough impairments in ND to sensory and cognitive, rather than motor, deficits [26]. Indeed, some irritant chemicals are known to readily elicit cough in ND patients with dystussia and atussia and have been used for decades in diagnostics and research, e.g., to measure cough reflex sensitivity in order to identify swallowing dysfunction or assess the risk of AP [5, 27–30]. A well-known example of such an irritant is capsaicin, the pungent extract of *Capsicum annuum* fruits (chili peppers), which, in aerosolized form, induces cough in a dose-dependent and safe manner. It also has good short- and long-term reproducibility [31, 32]. The ability of aerosolized capsaicin (AC) to induce cough in ND patients with cough impairments suggests that the potential to produce cough is present but “inaccessible” due to neurological disorder. We termed this unrealized potential “cough potential” (CP).

This study aimed to assess the use of AC as a therapeutic tool in treating ND-related cough impairments. For this, the cough response to AC was quantified. Spirometry was used to measure peak cough flow (PCF) of voluntary cough (cough on command) and reflexive, or AC-induced, cough. PCF is an indicator of cough efficacy in terms of its tracheobronchial clearance capabilities [33]. It ranges from 6 to 14 L/s in healthy individuals. Cough with a PCF above 4.5 L/s is defined as effective, and between 2.7 and 4.5 L/s as partially effective. Cough with a PCF below 2.7 L/s is considered ineffective and associated with increased risk of AP [34–36]. In addition, we introduced a novel application method that allows AC treatment to be performed at home by the patients themselves or their caregivers.

The a priori hypothesis was that in ND patients with dystussia and atussia, a CP is present but inaccessible due to neurological disorder. It was further hypothesized that AC would allow this patient population to access their individual CP and perform adequate tracheobronchial clearance.

## Methods

This study was designed as a controlled intervention study. Participants with ND were recruited from inpatients and outpatients diagnosed and treated for ND of various etiologies at Kliniken Valens, Valens, Switzerland. Controls without ND were recruited among volunteers. Data were collected between July 2016 and March 2017. All participants provided verbal and written informed consent. This study received ethics approval from the Ethikkommission Kanton St. Gallen, Switzerland (Project ID: 2016-00735; WHO ID: DRKS00010719). Ethical guidelines, as defined by the Declaration of Helsinki, were observed.

Spirometry was used to measure PCF of voluntary cough (voluntary PCF) and reflexive cough (reflexive PCF) secondary to pharyngeal exposure to AC in 30 people with ND and 30 controls without ND. Data were collected during single sessions. First, voluntary PCF was measured. The procedures were identical for both groups. All participants met inclusion criteria if they were non-smokers between 18 and 90 years of age.

Exclusion criteria for the ND group were chronic respiratory diseases (e.g., chronic obstructive pulmonary disease; COPD), endotracheal intubation, facial nerve palsy (inaccurate measurement due to difficulties tightly sealing the lips around the mouthpiece), acute cardiovascular diseases, and inability to comply due to cognitive or neuropsychological dysfunction (e.g., acute mental health disorders, dementia). Exclusion criteria for the control group were chronic respiratory diseases (e.g., chronic obstructive pulmonary disease; COPD), acute cardiovascular diseases, and swallowing impairments.

PCF data were obtained using a digital spirometer (EasyOne®, ndd Medizintechnik AG, Feuerthalen, Switzerland), which meets spirometry standards, as defined by the American Thoracic Society (ATS) and the European Respiratory Society (ERS) [37, 38]. Due to the location of Kliniken Valens, the spirometer was calibrated to 920 m mean sea level (MSL). A service representative performed the calibration and operating system update. All procedures were completed with participants sitting in an upright position on a chair or in a wheelchair at a table in a quiet clinic room. If needed, postural adjustments were made to ensure respiratory comfort. The PCF of three voluntary coughs (on command) and three reflexive coughs (secondary to pharyngeal exposure to AC) was measured. The primary outcome measure for both types of cough was the highest PCF value produced.

**Fig. 1:**
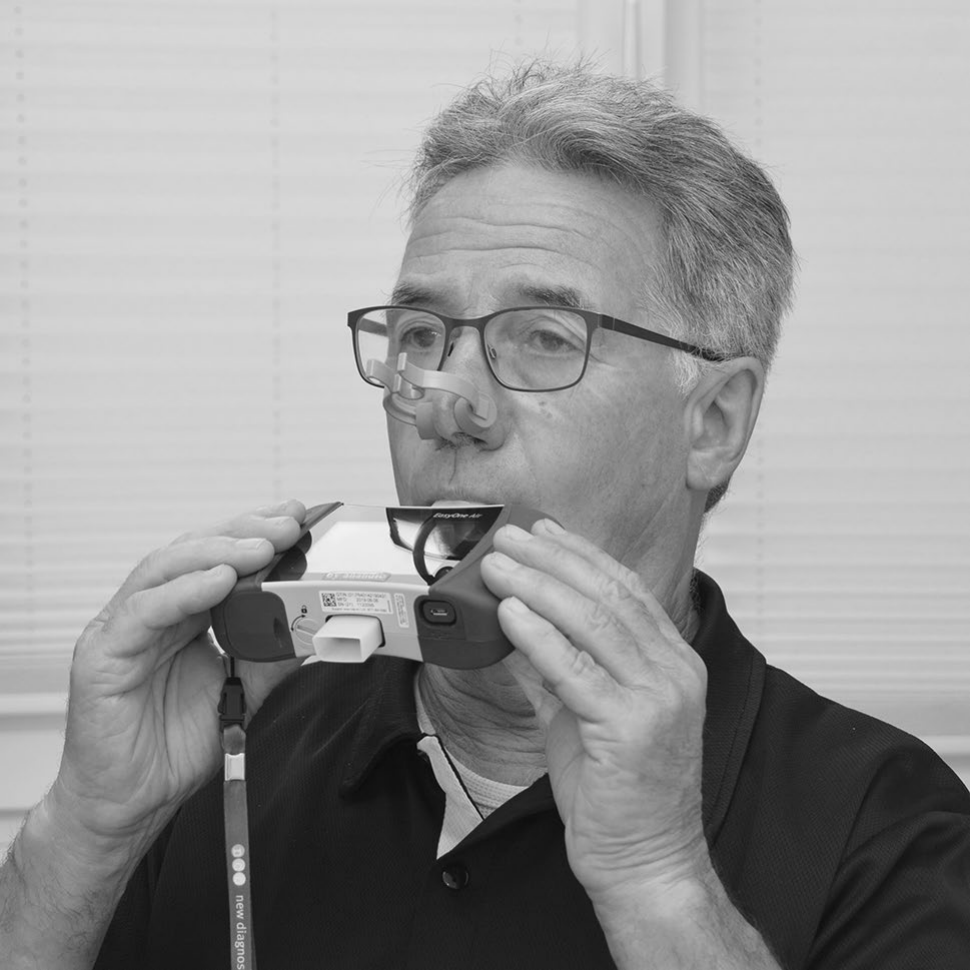
Patient during voluntary peak cough flow (PCF) measurement

Before testing started, participants were familiarized with the procedures and equipment. The EasyOne® spirometer is a lightweight device, ergonomically designed to be hand-held by the user. If a participant’s motor skills were restricted, the person applying the test provided assistance. If a participant’s restrictions were severe, medical staff familiar with the procedures provided additional support. To prevent air leaks, all participants wore a nose clip for both voluntary and reflexive PCF measurements. In addition, they were given detailed instructions on the correct use of the mouthpiece, i.e., how to seal the lips around it tightly. Verbal guidance was provided throughout the procedure. Measurement of voluntary PCF started with the person applying the test putting the nose clip in place and asking the participants to seal their lips tightly around the mouthpiece (Fig. 1). Participants were then instructed to “fully exhale, rapidly inhale to full capacity, and cough into the mouthpiece with maximum effort.”

Reflexive cough was induced via pharyngeal exposure to aerosolized capsaicin ((6E)-N- [(4-Hydroxy-3-methoxyphenyl)methyl]-8-methylnon-6-enamide). The capsaicin aerosol was generated by dissolving 1 drop of liquid cayenne extract (BioPräp, Osnabrück, Germany, 1.5–2% capsaicin, 500,000 Scoville Heat Units; SHU) in 100 mL cold (8 °C), carbonated water (0.00075–0.001% capsaicin). Disposable hard-plastic cups with 100 mL markings were used to ensure accurate dosing.

**Fig. 2:**
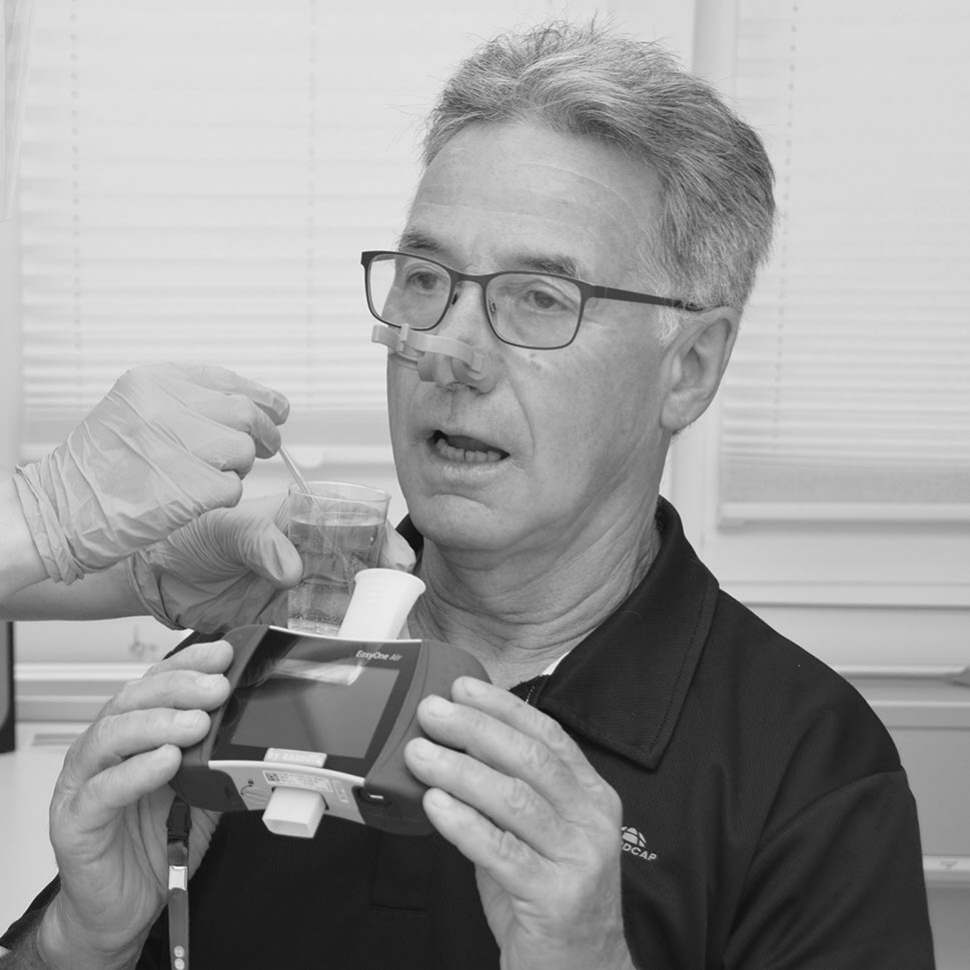
Patient during reflexive peak cough flow (PCF) measurement (secondary to pharyngeal exposure to aerosolized capsaicin); test person holds transparent hard-plastic cup with capsaicin solution (liquid cayenne extract, containing 1.5–2 s% capsaicin, dissolved in 100 mL carbonated water) 2–5 cm from the patient’s mouth at chin level and stirs the solution gently with a hard-plastic teaspoon

The test started with participants holding the spirometer with both hands (Fig. 2). The person applying the test then prepared the capsaicin solution as described above and moved the cup approximately 2–5 cm away from the participant’s mouth at chin level. While carefully stirring the sparkling solution with a hard-plastic teaspoon to facilitate aerosol production, the person applying the test asked the participants to “breathe in gently” (but not to inhale forcefully).

At the onset of the urge-to-cough, the participants immediately sealed their lips around the mouthpiece and directed the cough into the spirometer. If the solution failed to induce cough, the capsaicin concentration was increased in single-drop steps, up to a maximum of 10 drops of liquid cayenne extract per 100 mL carbonated water (0.0075–0.01% capsaicin). For each step, a new cup and fresh water were used. The spirometer stored all PCF measures automatically. After each session, the data were transferred to a secure server.

### Statistical Analysis

G*Power® software (Heinrich Heine Universität, Düsseldorf, Germany, version 3.1.) was used to calculate the sample size [39]. The minimum sample size required was 27 participants (80% power; desired effect size 0.5; alpha 0.05). SPSS® software (IBM®, Armonk, USA, version 24.0) was used for statistical analysis. Wilcoxon *t* test was used to compare voluntary and reflexive PCF within the groups. Comparisons between the two groups were made using Mann–Whitney *U* test. The significance level for all statistical tests was set at *p* < 0.05. A PCF of 4.5 L/s was considered effective [34]. In addition, analysis of covariance (ANCOVA) was performed to test the influence of sex, age, height, weight, and body mass index (BMI) on voluntary and reflexive PCF.

## Results

Table 1 summarizes the participants’ demographic information.

**Table 1:** Participants’ demographic information

	ND group	Control group
Participants; *n*	28	30
Age; mean years (SD)	64 (12)^a^	49 (15)^a^
Sex; *n* (%)	13 (46) female^a^	23 (77) female^a^
Weight; mean kg (SD)	69 (15)	64 (12)
Height; mean cm (SD)	171 (9)	167 (6)
BMI; mean kg/m^2^ (SD)	24 (5)	23 (4)

*SD* standard deviation, *ND* neurogenic dysphagia, *BMI* body mass index

^a^Indicates significant difference between the ND and control group (*p* < 0.001)

In terms of age and sex, there were statistically significant differences between the two groups, with the control group being younger and comprising primarily female participants. There were no significant differences in terms of weight, height, and BMI. Participants in the ND group had been diagnosed with multiple sclerosis (*n* = 16); stroke (*n* = 7); Parkinson’s disease (*n* = 3); traumatic brain injury (*n* = 2); multiple system atrophy (*n* = 1); and critical illness polyneuropathy (*n* = 1). Eight participants in the ND group (*n* = 1 with multiple system atrophy, *n* = 1 with traumatic brain injury, *n* = 1 with stroke, and *n* = 5 with multiple sclerosis) presented an extraordinarily strong reflexive cough response to AC. Therefore, they were unable to form a tight seal around the mouthpiece. However, according to the participants’ own assessment and the observations of the person applying the test (i.e., cough sounds, body flexions), reflexive PCF was noticeably higher than voluntary PCF. Therefore, missing data for reflexive PCF were imputed to the highest reflexive PCF measured in the ND group (7.73 L/s).

Cough threshold capsaicin concentration, i.e., the number of drops of liquid cayenne extract per 100 mL carbonated water required to induce reflexive cough, was significantly higher in the ND group than in the control group (*p* < 0.001). As shown in Table 2, the lowest concentration of 1 drop of liquid cayenne extract per 100 mL carbonated water induced cough in 11 participants with ND (39.3%) and 27 controls (90%). Two participants in the ND group (*n* = 1 with Parkinson’s disease and *n* = 1 with traumatic brain injury) did not cough when administered the maximum concentration of 10 drops per 100 mL carbonated water. They were not included in the statistical analysis. A complete analysis was performed on 28 participants with ND.

**Table 2:** Median and interquartile range (IQR) of cough threshold concentrations

	ND group	Control group
Capsaicin concentration eliciting cough; median of number of drops (IQR)	2 (1–3)	1 (1–1)
1 drop LCE/100 mL carbonated water	11 (39.3%)^a^	27 (90%)^a^
2 drops LCE/100 mL carbonated water	6 (21.4%)^a^	2 (6.7%)^a^
3 drops LCE/100 mL carbonated water	7 (25%)^a^	1 (3.3%)^a^
4–10 drops LCE/100 mL carbonated water	4 (14.3%)^a^	0 (0%)^a^

*LCE* Liquid cayenne extract (1.5–2% capsaicin), *IQR* Interquartile range, *ND* neurogenic dysphagia

^a^Number of participants *n* (%)

Table 3 shows that, in the ND group, reflexive PCF was significantly higher than voluntary PCF (*p* = 0.001). In 20 patients with ND (71.4%), voluntary PCF was below the value described in the literature as effective. In 8 patients with ND (28.6%), voluntary cough was effective. In 19 ND patients (67.9%), reflexive PCF was effective. It was partially effective in 5 ND patients (17.9%), and in 4 ND patients with multiple sclerosis (14.3%), it was ineffective. In other words, after pharyngeal exposure to AC, 24 of 28 patients with ND (85.8%) were able to produce an effective or partially effective cough.

**Table 3:** Correlation between peak cough flow (PCF) and tracheobronchial clearance efficacy; according to Bach et al. [16]

		ND group		Control group	
PCF (L/s)		Voluntary cough; *n* (%)	Reflexive cough; *n* (%)	Voluntary cough;* n* (%)	Reflexive cough; *n* (%)
> 4.5	Effective clearance	8 (28.6%)	19 (67.9%)	29 (96.7%)	26 (86.7%)
2.7–4.5	Partly effective clearance	7 (25%)	5 (17.9%)	1 (3.3%)	3 (10%)
< 2.7	Ineffective clearance	13 (46.4%)	4 (14.3%)	0 (0%)	1 (3.3%)

*PCF* peak cough flow, *ND* neurogenic dysphagia

In the control group, reflexive PCF was significantly lower than voluntary PCF (*p* < 0.001). Voluntary PCF was effective in 29 controls (96.7%) and partially effective in 1 control (3.3%), whereas reflexive PCF was effective in 26 controls (86.7%), partially effective in 3 controls (10%), and ineffective in 1 control (3.3%).

**Fig. 3:**
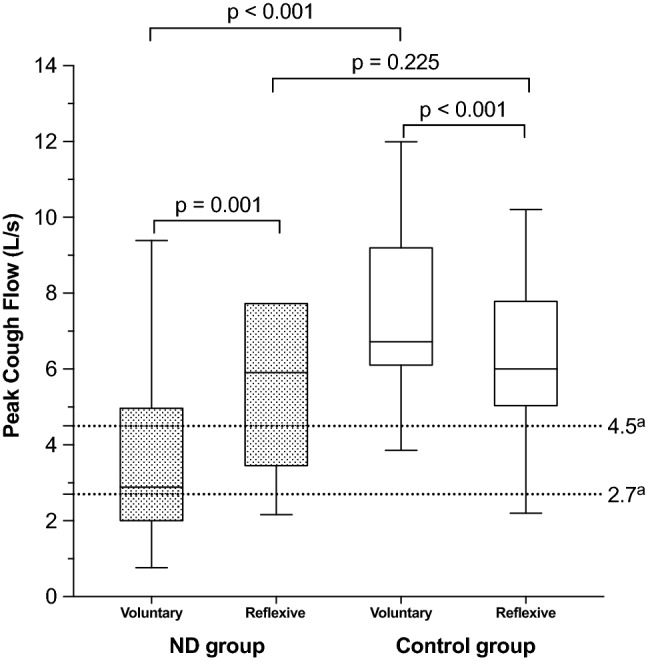
Boxplots of voluntary and reflexive peak cough flow (PCF) in patients with neurogenic dysphagia (ND) and healthy controls. The boxplots show the medians and interquartile ranges. The whiskers indicate the lowest and maximum measurements. ^a^Dotted lines for cut-off values: PCF > 4.5 L/s = effective clearance; PCF between 4.5 L/s and 2.7 L/s = partly effective clearance; PCF < 2.7 = ineffective clearance [16]

A graphic depiction of the ND versus the healthy control cohort's voluntary and reflexive PCF is shown in Fig. 3. Medians and interquartile ranges (IQR) are shown in Table 4. Voluntary and reflexive PCF were both higher in the control group than in the ND group (voluntary PCF: *p* < 0.001; reflexive PCF: *p* = 0.225).

**Table 4:** Median and interquartile range (IQR) of voluntary and reflexive peak cough flow (PCF)

	ND group	Control group
Voluntary cough; median PCF (IQR)	2.89 (2.00–4.96)	6.72 (6.11–9.20)
Reflexive cough; median PCF (IQR)	5.90 (3.45–7.73)	6.00 (5.04–7.79)

*PCF* peak cough flow (L/s), *IQR* interquartile range

Covariance analysis was used to evaluate factors influencing the difference between voluntary and reflexive PCF. After adjusting for age, sex, height, weight, and BMI, ND was the only significant determinant of the difference (*F* = 21.780; *p* < 0.001).

## Discussion

This study quantified the cough response to AC in ND, and assessed the potential of AC as a therapeutic tool in treating ND-related dystussia and atussia. The study also introduced a novel application method that allows AC treatment outside of the clinical setting.

As expected, voluntary PCF was significantly lower in the ND group compared with the control group. In the ND group, 29% of participants performed adequate tracheobronchial clearance on command, compared with 97% in the control group. However, secondary to pharyngeal exposure to AC, 68% of participants with ND performed adequate tracheobronchial clearance.

Like reflex cough in response to aspiration, reflex cough secondary to pharyngeal exposure to AC may be considered more ecologically valid than voluntary cough, as they are both induced by sensory stimuli [5]. Therefore, reflexive PCF would be expected to be higher than voluntary PCF in the control group. However, reflexive PCF was found to be significantly lower. Earlier studies have shown that reflexive cough is down-regulated in cortical and subcortical diseases, suggesting an involvement of suprapontine structures [40–42]. Hutchings and colleagues were the first to demonstrate that the cough threshold to a tussigenic stimulus was significantly higher when participants were instructed “not to cough,” i.e., to suppress cough [43]. Cortical down-regulation via the somatosensory pathway (urge-to-cough) as a reaction to the strong stimulus may have contributed to this unexpected result. It is possible that, with more precise verbal instructions from the person applying the test, such as “not to interfere with the cough response,” cortical down-regulation could have been prevented.

Threshold capsaicin concentrations required to induce cough ranged from 1 drop of liquid cayenne extract per 100 mL carbonated water to 10 drops. The lowest capsaicin concentration induced cough in 90% of healthy controls, whereas participants with ND required significantly higher concentrations (Table 2). A significant correlation between cough threshold capsaicin concentrations and dysphagia severity has been described elsewhere [36].

AC does not make cough stronger. It merely enables ND patients with dystussia and atussia to access their CP, which we hypothesize is present, but difficult to access or inaccessible due to neurological disorder. Hence, patients with ND and muscle weakness (e.g., amyotrophic lateral sclerosis; ALS) may not benefit from AC treatment as their cough impairments are caused by motor deficits.

The literature suggests that capsaicin activates TRPV1 receptors, which are known to participate in pain perception and cough induction [44, 45]. In this study, the capsaicin aerosol was not forcefully inhaled, but rather gently breathed in. Therefore, the involvement of sensory receptors located below the larynx can be ruled out. In addition, we know from clinical practice that systemic application of a capsaicin solution per os (1 drop of liquid cayenne extract dissolved in 100 mL still water) temporarily inhibits cough induction with AC by desensitizing the participating receptors. This suggests the involvement of TRPV1-expressing C-fibers located in the pharynx [46]. However, much is yet to be understood about the underlying mechanisms of reflex cough secondary to pharyngeal exposure to AC.

AC generated by a nebulizer is considered pH neutral. In this study, carbonated water was used as a vehicle solution for capsaicin aerosol generation. The aerosol could therefore be expected to be slightly acidic. However, pre-study experiments have shown that pharyngeal exposure to the aerosol generated with carbonated water does not induce cough. Therefore, AC’s pH level was not considered in this study.

Clinically, airway clearance is considered incomplete if patients cannot remove the aspirated material permanently by correctly swallowing or expectorating it. A post-cough swallow was observed in the current study, which, to our knowledge, has not been described previously in the literature. It appears to be reflexively induced at the end of a cough epoch and generally more effective than a regular swallow. Even in ND patients with severe aspiration, this single post-cough swallow almost always allows for successful permanent removal of aspirated material.

Capsaicin aerosol generation and application usually require highly sophisticated equipment [28]. This may be less of a problem for researchers and diagnosticians in specialized laboratories and hospitals, where such equipment is readily available. However, the lack of proper equipment would pose a significant problem when considering using AC therapeutically. Therefore, we developed an application method that allows AC treatment at home, either by the patients themselves or their caregivers.

After introducing AC into our own clinical practice, an increasing number of clinics in Switzerland and Germany added AC as a therapeutic tool to their ND toolbox. Capsaicin is a widely used substance. A vast amount of literature has documented the various application methods in terms of their safety and efficacy. The often highly medicated ND patients appreciate that capsaicin is a natural substance whose origin and effect they understand. The same is true for their families and caregivers. Traditional ND treatment focusing on aspiration prevention is associated with increased anxiety and fear of aspiration. Introducing aspiration correction into ND treatment and AP prevention helps alleviate that fear. Since AC requires minimal patient cooperation, even neurologically severely ill individuals may benefit from the treatment. In our clinical practice, it is not unusual that AC helps nihil per os patients (Penetration–Aspiration Scale (PAS) 7 and 8) return to at least minimal food intake per os. The extent to which patients benefit from AC is highly individual. Therefore, the use of AC should be assessed on a case-by-case basis. To our knowledge, this is the first study to investigate the therapeutic use of AC in ND patients with dystussia and atussia.

## Limitations

This study has some limitations. The ND and control groups were not matched for age and sex distribution. However, they were well matched for height, weight, and BMI. Furthermore, ND etiologies were not considered in the statistical analysis. To consolidate the current findings, future studies should include larger patient groups and subgroups. Participants were not screened for angiotensin-converting enzyme (ACE) inhibitors, which are known to affect the cough response to a stimulus [47]. AP pathogenesis is multifactorial and complex. Other contributing factors, such as consistency, size, and type (pH level) of the bolus, were not considered in the study. Cough efficacy threshold values available in the literature are derived from Bach et al. ’s 1996 study, which concluded that the ability to generate a PCF of at least 160 L/min (2.7 L/s) is necessary for successful extubation or tracheostomy tube decannulation of patients with neuromuscular disease [34]. Although these values are widely used to predict cough efficacy in diagnostics and research, they are not validated for patients with ND and increased cough threshold. Further research is needed to establish validated and standardized cough efficacy threshold values that include, similar to peak expiratory flow (PEF), sex, age, height, weight, and other contributing factors. The novel application method presented in this study requires the use of liquid cayenne extract, whose capsaicin concentration is variable. Therefore, cough threshold concentration measurements may be less accurate than measured with a nebulizer and should be regarded as a limitation.

## Conclusion

In ND, the critical factor for AP pathogenesis may not be aspiration per se, but dystussia and atussia. This study shows that AC treatment allows ND patients to access their individual CP, and perform adequate tracheobronchial clearance. AC treatment is safe, even for neurologically severely ill patients, and requires only minimal patient cooperation. In addition, the application method described in this study allows for the continuation of AC treatment at home. The results of this study suggest that AC treatment may be a valuable therapeutic tool that should be considered as a new addition to the ND toolbox. However, further research is needed to assess the impact of AC treatment on AP prevention and patients' quality of life. This includes the necessity of making the clearance effect of AC on the airway visible, e.g., using fiberoptic evaluation of swallowing (FEES). Capsaicin should continue to gain acceptance as a therapeutic tool and play an essential role in the design of innovative treatment concepts.
